# The 2017 ACR TI-RADS: pictorial essay

**DOI:** 10.1590/0100-3984.2020.0141

**Published:** 2022

**Authors:** André Tojal Pires, Amina Muhamad Mota Mustafá, Márcio Olavo Gomes Magalhães

**Affiliations:** Department of Radiology and Diagnostic Imaging, Hospital Universitário de Brasília, Brasília, DF, Brazil.

**Keywords:** Thyroid gland, Thyroid diseases, Ultrasonography, Glândula tireoide, Doenças da tireoide, Ultrassonografia

## Abstract

High-resolution ultrasound is the imaging method of choice for the evaluation of thyroid nodules. The method has recently come to be used widely and often, which has increased the rate of thyroid nodule detection. In 2017, the American College of Radiology (ACR) established a risk-stratification system designated the Thyroid Imaging Reporting and Data System (TI-RADS) to be a practical guide for widespread use, with a single lexicon and standardization of ultrasound reports of thyroid nodules. The objective of this study was to present a practical approach, using examples to illustrate the criteria evaluated by the 2017 ACR TI-RADS, in order to help minimize uncertainties regarding its application by sonographers.

## Abstract (pt)

A ultrassonografia de alta resolução é a modalidade de escolha para avaliação de imagem dos nódulos tireoidianos, e sua recente aplicação ampla e difusa tornou a detecção de nódulos tireoidianos mais frequentes. O American College of Radiology (ACR) estabeleceu um sistema de estratificação de risco denominado Thyroid Imaging, Reporting and Data System (TI-RADS) para ser um guia prático para utilização ampla com um léxico único e padronização de relatórios ultrassonográficos de nódulos tireoidianos. O objetivo deste trabalho é fazer uma abordagem prática com base em exames para ilustrar e exemplificar os critérios avaliados pelo TI-RADS-ACR 2017, a fim de ajudar a reduzir os pontos de dúvidas de sua aplicação pelos profissionais ultrassonografistas.

## INTRODUCTION

High-resolution ultrasound is the imaging method of choice for the evaluation of thyroid nodules. The method has recently come to be used widely and often, which has increased the rate of thyroid nodule detection. Although the prevalence of thyroid nodules is high, the incidence of malignancy is relatively low in incidental nodules(^1^,^2^).

The diagnosis of malignancy depends, above all, on fine-needle aspiration biopsy or excisional biopsy. To avoid unnecessary procedures, risk stratification through systematization is essential^(3)^. In 2017, the American College of Radiology (ACR) convened to establish a practical risk stratification system for widespread use by all medical professionals, resulting in the Thyroid Imaging Reporting and Data System (TI-RADS), which provides a single lexicon to reduce confusion in ultrasound reports of thyroid nodules(^4^,^5^,^6^).

The objective of this study was to create a practical guide to help minimize uncertainties regarding the application of the 2017 ACR TI-RADS by sonographers. To that end, we use examples to illustrate the ACR TI-RADS criteria and scoring.

### FEATURES EVALUATED

The ACR TI-RADS is based on the evaluation of five key features of a nodule—composition, echogenicity, shape, margin, and echogenic foci—which are scored individually, the feature scores being summed to arrive at the final classification of the risk level, which ranges from TR1 (benign) to TR5 (highly suspicious for malignancy). A sixth feature (size) is used in order to determine the appropriate course of action. For each of the five key features, one of the options must be chosen and duly scored, with the exception of the “echogenic foci” feature, in which all the options applicable to the evaluated node must be described and scored^(4)^. According to the lexicon proposed by the ACR TI-RADS, the features described above will be designated in categories.

### COMPOSITION

**Figure 1:**
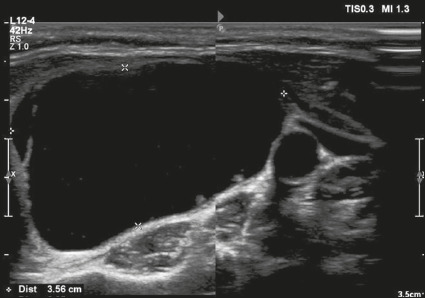
Image of a nodule that was completely cystic. Nodules that are completely cystic, predominantly cystic, or spongiform are not scored for other categories, therefore automatically receiving a final score of 0 and classified as TR1.

The composition of a nodule is defined on the basis of its content (solid tissue or fluid). That content is classified as detailed below. **Cystic** – This describes a nodule that is completely or almost completely filled with fluid (Figure 1).

**Spongiform** – This describes a nodule that is composed of multiple small cystic spaces that occupy at least 50% of the total volume of the nodule.

**Figure 2:**
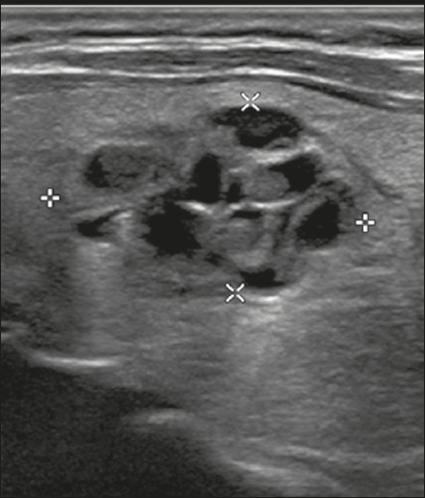
Image of a mixed solid-cystic nodule. In such nodules, only the solid component should be scored for the echogenicity, margin, and echogenic foci categories. In this case, the nodule was assigned 1 point for being mixed, 2 points for being hypoechoic, 0 points for being wider-than-tall, 0 points for having undefined margins, and 0 points for having no acoustic shadowing artifacts or echogenic foci. Therefore, the total score was 3 points and the risk level was classified as TR3.

**Mixed solid-cystic** – This describes a nodule that combines two features presented in the original lexicon (predominantly solid and predominantly cystic)^(4)^. In the evaluation of these nodules, the characterization of the solid component is more important than the proportional distribution of the solid and cystic components (Figure 2).

**Figure 3:**
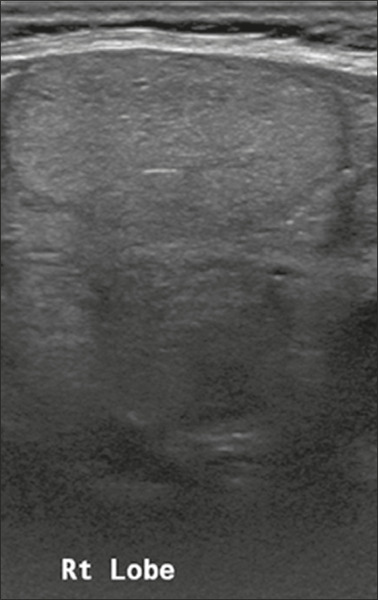
Image of a completely solid nodule, with echogenicity similar to the rest of the thyroid parenchyma, presenting a hypoechoic halo that should not be scored for the echogenicity or margin categories. The features of (scores for) this nodule were as follows: solid (2 points); isoechoic (1 point); wider-than-tall (0 points); smooth margins (0 points); and no acoustic shadowing artifacts or echogenic foci (0 points). Therefore, the total score was 3 points and the risk level was classified as TR3.

**Solid** – This describes a nodule that is completely or almost completely composed of soft tissue (Figure 3). There is no precise definition of the proportion of solid component required for a nodule to be classified as solid, which is often a subjective finding. As a general rule, nodules that are mostly solid and contain cystic spaces accounting for no more than 5% of their total volume are classified as solid^(5)^. In some cases, it can be difficult to distinguish between solid content and debris/hemorrhagic material. In such cases, the use of color Doppler can help identify flow within the solid component. If the composition of a nodule cannot be determined, it should be considered to be solid.

### ECHOGENICITY

The echogenicity of a nodule is graded in relation to the adjacent tissue (thyroid parenchyma or anterior cervical musculature). Note that, for this category, only the solid component should be taken into account.

**Figure 4:**
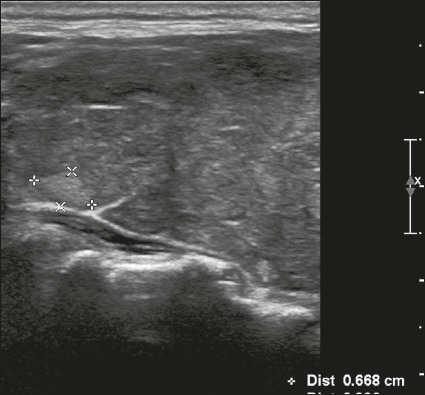
Image of a hyperechoic nodule. The features of (scores for) this nodule were as follows: solid (2 points); hyperechoic (1 point); wider-than-tall (0 points); smooth margins (0 points); and no acoustic shadowing artifacts or echogenic foci (0 points). Therefore, the total score was 3 points and the risk level was classified as TR3.

**Figure 5:**
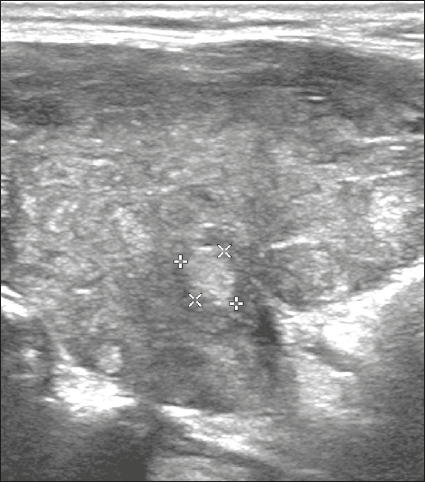
Image of a hyperechoic nodule. Note the heterogeneous echotexture of the thyroid parenchyma, especially the presence of nodules with well-defined margins and echogenicity greater than that of the rest of the parenchyma. The nodule pictured was solid (2 points), hyperechoic (1 point), and wider-than-tall (0 points), with smooth margins (0 points) and without acoustic shadowing artifacts or echogenic foci (0 points). Therefore, the total score was 3 points and the risk level was classified as TR3.

**Hyperechoic** – This describes a nodule with increased echogenicity in relation to the thyroid parenchyma (Figures 4 and 5).

**Isoechoic** – This describes a nodule with echogenicity similar to that of the thyroid parenchyma (Figure 3). If the echogenicity of a nodule cannot be determined, it should be considered isoechoic for scoring.

**Figure 6:**
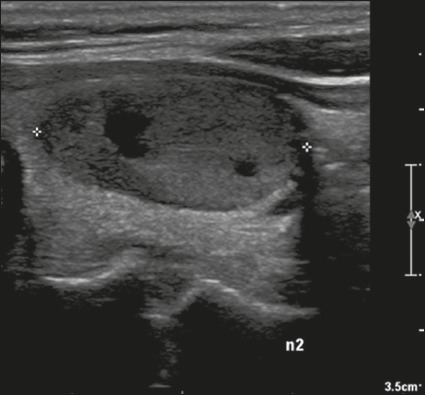
Image of a predominantly solid nodule with smooth margins that is less echogenic than the rest of the thyroid parenchyma. The nodule pictured was solid (2 points), hypoechoic (2 points), and wider-than-tall (0 points), with well-defined margins (0 points) and without acoustic shadowing artifacts or echogenic foci (0 points). Therefore, the total score was 4 points and the risk level was classified as TR4.

**Figure 7:**
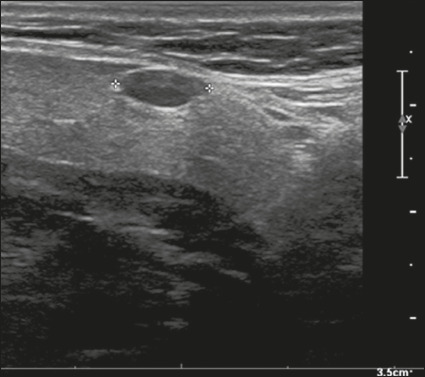
Image of an oval-shaped nodule with well-defined margins and echogenicity lower than that of the rest of the thyroid parenchyma. In this case, the nodule was assigned 2 points for being solid, 2 points for being hypoechoic, 0 points for being wider-than-tall, 2 points for having a lobulated margin, and 0 points for having no acoustic shadowing artifacts or echogenic foci. Therefore, the total score was 6 points and the risk level was classified as TR4.

**Hypoechoic** – This describes a nodule with reduced echogenicity in relation to the thyroid parenchyma (Figures 6 and 7).

**Figure 8:**
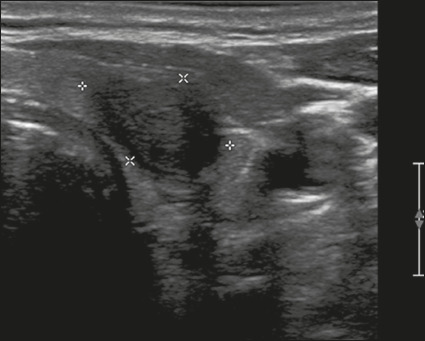
Image of a markedly hypoechoic nodule. Compare the echogenicity of the nodules with that of the cervical musculature. Attention should be paid to the ultrasound parameters. The nodule pictured was solid (2 points), markedly hypoechoic (3 points), and wider-than-tall (0 points), with undefined margins (0 points) and without acoustic shadowing artifacts or echogenic foci (0 points). Therefore, the total score was 5 points and the risk level was classified as TR4.

**Markedly hypoechoic** – This describes a nodule with reduced echogenicity in relation to the anterior cervical musculature (Figure 8). This characteristic is highly specific for malignancy.

### SHAPE

In the shape category, only one aspect of a nodule is evaluated for ACR TI-RADS risk stratification: the relationship between its anteroposterior dimension (tallness) and its transverse dimension (width) on an axial image. A nodule is classified as either wider-than-tall or taller-than-wide.

**Figure 9:**
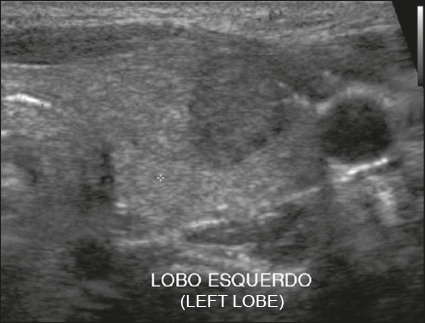
Image of a predominantly solid, hypoechoic, taller-than-wide nodule with well-defined margins. The features of (scores for) this nodule were as follows: solid (2 points), hypoechoic (2 points), taller-than-wide (3 points), smooth margins (0 points), and no acoustic shadowing artifacts or echogenic foci (0 points). Therefore, the total score was 7 points and the risk level was classified as TR5.

**Taller-than-wide** – When the anteroposterior dimension of a nodule is greater than its transverse dimension (Figure 9), it is classified as taller-than-wide. Although not very sensitive, this is a finding that id highly specific for malignant lesions, especially when found in combination with other aspects suggestive of malignancy.

### MARGIN

The margin category classifies the interface between a nodule and the adjacent intrathyroidal or extrathyroidal tissue.

**Smooth** – This describes nodule margins that are well-defined, curved, and uninterrupted (Figure 3).

**Figure 10:**
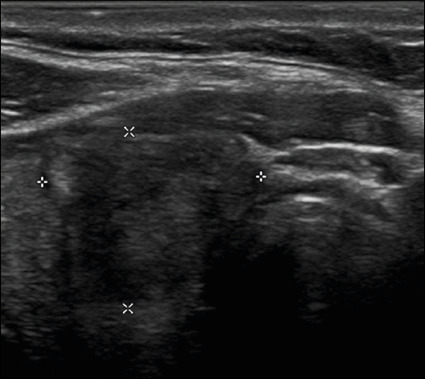
Image of a solid nodule with lobulated margins and a rounded lobulation in its anterior portion. In this case, the nodule was assigned 2 points for being solid, 2 points for being hypoechoic, 0 points for being wider-than-tall, 2 points for having a lobulated margin, and 0 points for having no acoustic shadowing artifacts or echogenic foci. Therefore, the total score was 6 points and the risk level was classified as TR4.

**Figure 11:**
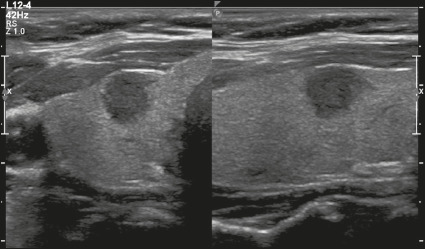
Image of a solid nodule with irregular margins. Note the irregularity, with an acute angle at the medial margin of the nodule. The nodule pictured was solid (2 points), hypoechoic (2 points), and wider-than-tall (0 points), with irregular margins (2 points) and without acoustic shadowing artifacts or echogenic foci (0 points). Therefore, the total score was 6 points and the risk level was classified as TR4.

**Irregular or lobulated** – This describes nodule margins that are spiculated and jagged, forming acute angles. Such a nodule may or may not have well-defined soft tissue protrusions into adjacent tissues (Figures 10 and 11).

**Figure 12:**
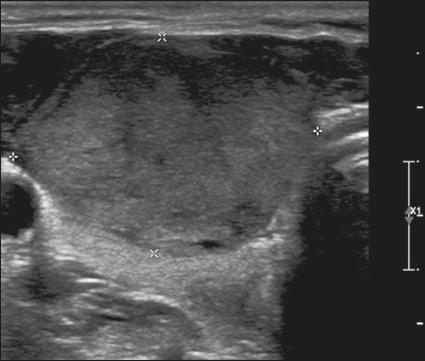
Image of a nodule extending beyond the anterior limit of the thyroid. The nodule pictured was solid (2 points), hypoechoic (2 points), and wider-than-tall (0 points), with extrathyroidal extension (3 points) and without posterior attenuation artifacts or echogenic foci (0 points). Therefore, the total score was 7 points and the risk level was classified as TR5.

**Extrathyroidal extension** – This describes nodule margins that extend beyond the limits of the thyroid gland, characterized by clear invasion of adjacent soft tissues or vascular structures (Figure 12).

**Figure 13:**
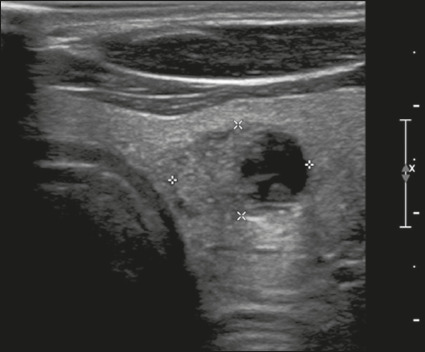
Image of a mixed solid-cystic nodule. Note that the medial margin of the nodule cannot easily be distinguished from the rest of the parenchyma. In this case, the nodule was assigned 1 point for being mixed, 2 points for being hypoechoic, 0 points for being wider-than-tall, 0 points for having ill-defined margins, and 0 points for having no acoustic shadowing artifacts or echogenic foci. Therefore, the total score was 3 points and the risk level was classified as TR3.

**Poorly defined or undefined** – This describes nodule margins that are difficult to distinguish from the thyroid parenchyma (Figure 13), without irregularities or spicules.

Although margins that are irregular or lobulated are suspicious for malignancy, ill-defined margins have not been statistically associated with malignant nodules and are quite common in benign hyperplastic nodules.

### ECHOGENIC FOCI

Echogenic foci are defined as focal areas of significantly increased echogenicity, which can vary in shape and size, within a nodule. They can be found in isolation or in combination with artifacts related to acoustic shadowing.

**Figure 14:**
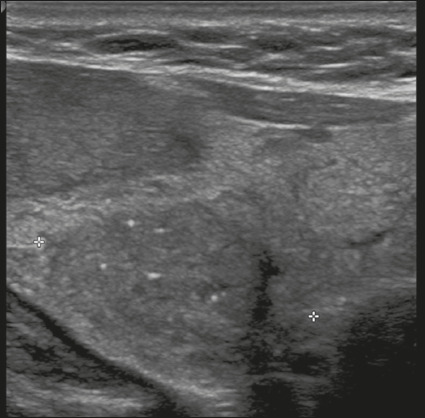
Image of a solid nodule, showing punctate echogenic foci. The nodule pictured was solid (2 points), hypoechoic (2 points), and wider-than-tall (0 points), with undefined margins (0 points) and punctate echogenic foci (3 points). Therefore, the total score was 7 points and the risk level was classified as TR5.

**Punctate echogenic foci** – These are defined as small echogenic spots without acoustic shadowing (Figure 14).

**Figure 15:**
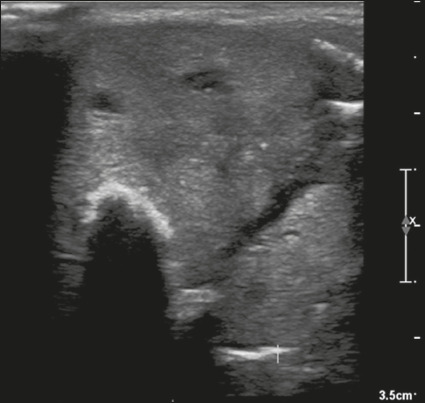
Image of a nodule with macrocalcification. Note the intense acoustic shadowing. The features of (scores for) this nodule were as follows: solid (2 points); isoechoic (1 point); wider-than-tall (0 points); smooth margins (0 points); and macrocalcification (1 point). Therefore, the total score was 4 points and the risk level was classified as TR4.

**Macrocalcifications** – These are defined as calcifications that are large enough to generate acoustic shadowing and can be irregular in shape (Figure 15).

**Figure 16:**
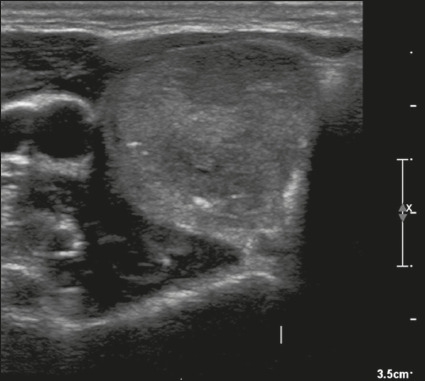
Image of a nodule with peripheral echogenic foci corresponding to calcifications. The nodule pictured was solid (2 points), hypoechoic (2 points), and wider-than-tall (0 points), with well-defined margins (0 points) and peripheral calcifications (2 points). Therefore, the total score was 6 points and the risk level was classified as TR4.

**Figure 17:**
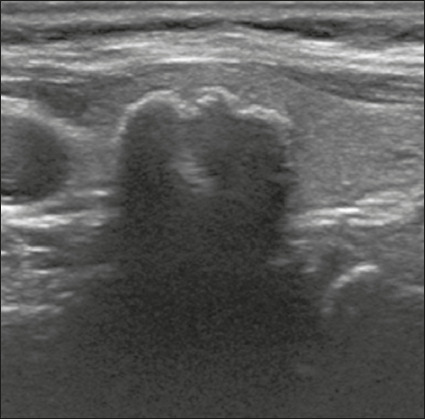
Image of a nodule with peripheral calcifications and acoustic shadowing that obscures its central content. According to the ACR TI-RADS, when the internal characteristics of a nodule cannot be determined because of acoustic shadowing, it is prudent to assume that it is solid and to assign it 2 points for composition, as well as 1 point for echogenicity. In this case, the nodule was assigned 2 points for being of indeterminate composition, 1 point for being of indeterminate echogenicity, 0 points for being wider-than-tall, 2 points for having lobulated margins, and 2 points for having peripheral calcifications. Therefore, the total score was 7 points and the risk level was classified as TR5.

**Peripheral (rim) calcifications** – These are defined as calcifications that occupy the periphery of the nodule and can be continuous or discontinuous (Figures 16 and 17). They usually produce acoustic shadowing that obscures the central content of the nodule.

### PRACTICAL EXAMPLES

**Figure 18:**
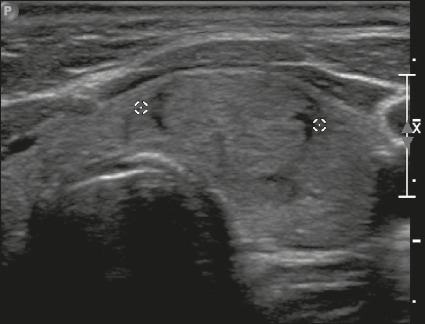
Image of a nodule that was solid (2 points), isoechoic (1 point), and wider-than-tall (0 points), with smooth margins (0 points) and without echogenic foci or acoustic shadowing artifacts (0 points). Therefore, the total score was 3 points and the risk level was classified as TR3.

Here, we provide examples of the practical application of the ACR TI-RADS (Figures 18^19^,20,21). The captions detail the items evaluated and show the scores in parentheses.

**Figure 19:**
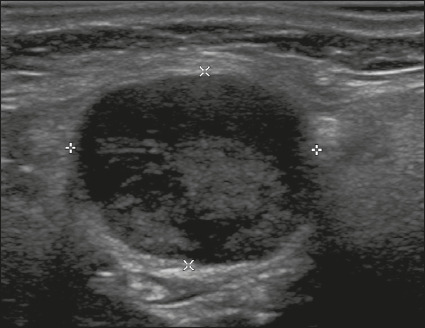
Image of a nodule that was solid (2 points), markedly hypoechoic (3 points), and wider-than-tall (0 points), with smooth margins (0 points) and without echogenic foci or acoustic shadowing artifacts (0 points). Therefore, the total score was 5 points and the risk level was classified as TR4.

**Figure 20:**
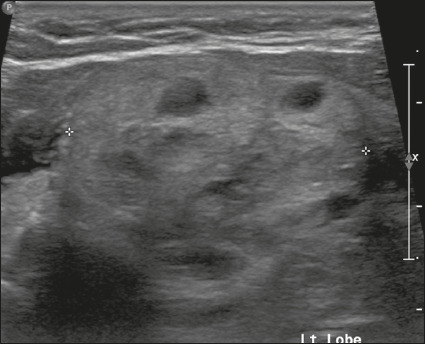
Image of a mixed solid-cystic nodule (1 point) that was isoechoic (1 point), was wider-than-tall (0 points), and extended beyond the anterior limit of the thyroid gland (3 points), without echogenic foci or acoustic shadowing artifacts (0 points). Therefore, the total score was 5 points and the risk level was classified as TR4.

**Figure 21:**
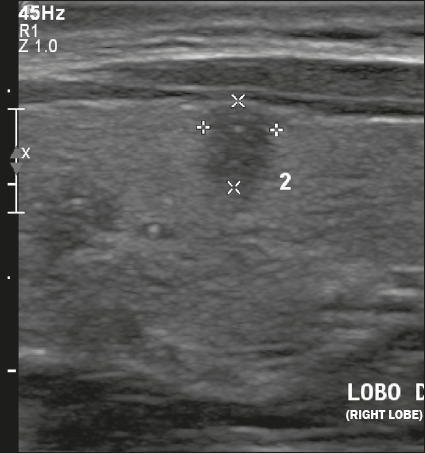
Image of a nodule that was solid (2 points), hypoechoic (2 points), and taller-than-wide (3 points), with undefined margins (0 points) and without acoustic shadowing artifacts or echogenic foci (0 points). Therefore, the total score was 7 points and the risk level was classified as TR5.

## DISCUSSION

Ultrasound is the main imaging method for evaluating thyroid nodules, and its wide availability has allowed such nodules to be detected at increasingly higher rates^(5)^. Therefore, various risk-stratification models have been developed, each with its own set of recommendations, which has created confusion among medical professionals. The ACR TI-RADS was developed with the objective of standardizing the description of and approaches to thyroid nodules, its practical form making it highly reproducible.

The application of the ACR TI-RADS has limitations. First, the use of a point-based system limits the assessment of scored items and does not take into account variables that may have different implications, such as the appearance of the solid component of a solid-cystic nodule; certain aspects of that appearance are known to be associated with malignancy but are not scored in the ACR TI-RADS(^6^,^7^). The system also has some pitfalls, which can erroneously increase the risk level and indicate biopsies unnecessarily. For example, the tiny punctate hyperechoic foci that represent the standard pattern for the normal parenchyma can be misinterpreted as echogenic foci^(4)^. In addition, the ACR TI-RADS is of limited utility in the evaluation of thyroid glands with multiple nodules, such as multinodular goiters, in which the gland is supplanted by multiple confluent nodules of similar appearance. Although malignancy cannot be definitively excluded under these conditions, it would be impracticable to biopsy each of these nodules. Therefore, the use of the ACR TI-RADS risk-stratification model is not practical in that scenario^(4)^.

Despite its limitations, the ACR TI-RADS has a place in clinical practice, although it must be well understood in order to be used effectively. It provides a standardized lexicon and facilitates the appropriate management of all thyroid nodules, making it possible to avoid unnecessary diagnostic procedures, thus reducing patient discomfort and health care costs(^6^,^7^).
